# Identification of the key genes and characterizations of Tumor Immune Microenvironment in Lung Adenocarcinoma (LUAD) and Lung Squamous Cell Carcinoma (LUSC)

**DOI:** 10.7150/jca.42531

**Published:** 2020-06-16

**Authors:** Lemeng Zhang, Jianhua Chen, Tianli Cheng, Hua Yang, Haitao Li, Changqie Pan

**Affiliations:** Thoracic Medicine Department 1, Hunan Cancer Hospital, Changsha, Hunan Province, P.R. China, 410013.

**Keywords:** Immune microenvironment, Lung adenocarcinoma, Lung squamous cell carcinoma, Tumor mutation burden, differentially expressed genes

## Abstract

This study aimed to investigate the key genes and immune microenvironment involved in different TNM stages of lung adenocarcinoma (LUAD) and lung squamous cell carcinoma (LUSC). The gene expression and clinical characteristics data were downloaded from the genomic data commons (GDC) database. After initial data processing, the characteristics of the immune microenvironment were analyzed. The differentially expressed genes (DEGs) in tumor vs. normal, and in early vs. advanced stages were screened, followed by Spearman correlation test for tumor infiltrating immune cells (TIICs) to identify immune-related genes. Finally, functional enrichment, protein-protein interaction, and survival analyses were performed. In LUAD, early stage was with higher immune scores, greater number of memory B cells and M0 macrophages compared to advanced stage. M0 and M2 macrophages, and resting memory CD4+ T cells accounted for a large proportion of TIICs in LUAD. The abundance of M0 macrophage infiltration was significantly correlated with the TNM stage and survival. In LUSC, early stage was with higher cytolytic activity and neoantigen burden compared to advanced stage. M0 and M2 macrophages, and plasma cells accounted for a large proportion of TIICs in LUSC. The abundance of resting and activated mast cells was significantly correlated with TNM stage, while resting dendritic cells, eosinophils, activated memory CD4 T cells, and mast cells were significantly correlated with prognosis. Tumor mutation burden analysis revealed that the median of variants per sample decreased from stage I to IV in LUAD, while it increased in LUSC. Further, 83 and 9 immune-related DEGs were identified in LUAD and LUSC, respectively, of which 23 genes in LUAD and 2 genes in LUSC correlated with survival. In conclusion, we identified the key genes, and characterized the tumor immune microenvironment in LUAD and LUSC which may provide therapeutic targets for the treatment of NSCLC.

## Introduction

Lung cancer remains the leading cause of cancer-related deaths worldwide 1. Non-small cell lung cancer (NSCLC) accounts for approximately 85% of lung cancer and the 5-year survival range from 73% in stage IA to 15% in stage IV 2. Besides clinical TNM stage, the histologic type and differentiation are also important factors associated with the prognosis 3. Lung adenocarcinoma (LUAD) and lung squamous cell carcinoma (LUSC) are the two major histologic subtype of NSCLC 4.

The therapeutic prospects for advanced NSCLC have changed remarkably with a deeper understanding of tumor and immune cells interactions and the development of immunological checkpoint inhibitors 5, 6. The immune surveillance can be co-opted by tumor cells to escape immune destruction 7, 8. Taube et al. revealed that PD-L1 (a ligand of programmed death-1, PD-1) was expressed by cancer cells and immune infiltrating cells and its expression reflected an immune-active microenvironment 9. The differences in recruitment and localization of immune cells in the tumor environment may represent different therapeutic and prognostic values 10. High density of mature dendritic cells and CD8+ T lymphocytes were found to be related to an improved outcome in NSCLC 11.

The occurrence and development of lung cancer is a complex and dynamic process that relies on the synergy between gene mutations and tumor microenvironment 12. The abundant communications among genes, proteins, as well as cells within tumor microenvironments, are the resource for biomarkers and targets in the diagnosis and treatment of lung cancer 10. The modification and interaction of genes in the immune microenvironment among different histological types and clinical stages of lung cancer will provide a systematic therapeutic perspective for lung cancer. However, the immune microenvironment of NSCLC in different clinical stage and histological type has not been fully understood. Therefore, further comprehensive analysis of the tumor immune microenvironment is urgently needed.

However, it is difficult and laborious to identify potential therapeutic targets and biomarkers by comprehensively and systematically profiling various immune cells from heterogeneous tumor samples based only on experiments. Bioinformatics approaches can directly extract cell-type specific information using sophisticated computational approaches 13. Studies of the tumor immune microenvironment increasingly involve complex datasets which mainly depend on sophisticated computational methods 14. The bioinformatics approaches were used in our study to investigate the immune microenvironment and the genes involved in different stages of LUAD and LUSC. Based on the gene expression and clinical data in genomic data commons (GDC, https://gdc.cancer.gov) database, the immune microenvironment characteristics, including immune score, cytolytic activity score, tumor mutation burden (TMB), neoantigens, and immune cells infiltration were investigated and the immune-related differential expressed genes (DEGs) were identified and analyzed. This study will provide a comprehensive perspective for the treatment of LUAD and LUSC.

## Materials and Methods

### Data acquisition and preprocessing

The RNA-seq fragments per kilobase million (FPKM), including corresponding clinical phenotype data of the two histologic subtype of NSCLC, including LUAD and LUSC were downloaded from the GDC database. The platform for the RNAseq data was Illumina HiSeq 2000 RNA Sequencing platform, and all the data were downloaded in August 2019. The data were re-annotated based on the annotation information (hg39, V22) in Gencode (https://www.gencodegenes.org/) database 15. The Ensembl_ID was converted into Symbol_ID, and the Ensembl_ID with the highest expression value was selected when multiple Ensembl_IDs matched the same Symbol_ID.

### Immune microenvironment analysis

Based on the formula,


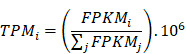


the gene expression data in FPKM format were converted into transcripts per million (TPM), which were then used in the analysis of immune microenvironment. The immune scores were calculated using the ESTIMATE (version 1.0.13) 16 in R package. The immune cytolytic activity score was calculated by the log-average of GZMA and PRF1 expression values in TPM 17. Then, the TMB was analyzed using the Maftools (version 2.0.16) 18 in R package based on the somatic mutation files called by using muTect software, followed by plotting of summary and oncoplots. Information about the neoantigens and neoantigen origin protein were downloaded from The Cancer Immunome Atlas (TCIA, https://tcia.at/home) database. The amount of neoantigens and neoantigen origin protein for each sample were counted, followed by standardization by log10. The *t* test was performed on the difference of immune scores, immune cytolytic activity score, neoantigens and neoantigen origin protein between tumor stages, and boxplot was created using ggpubr (version 0.2.2)^19^ in R package. The tumor infiltrating immune cells (TIICs) in LUAD and LUSC were analyzed based on the CIBERSORT algorithm 20, to estimate the abundance value of 22 immune cell types between different tumor stages.

### Identification of immune-related DEGs

After filtering the genes with low expression values (genes with a count of 0 in more than 50 % samples), the differentially expressed analysis between early stages and advanced stages were performed for the tumor samples with clinical stages information. The differentially expressed analysis of tumor vs. normal were also performed using limma package 21 (version: 3.40.6) after data preprocessing using edgeR (version: 3.26.6) 22. The DEGs were screened with the threshold of P value < 0.05 and |log_2_ fold change (FC)| > 0.585, and the volcano plot and heatmaps were plotted using ggplot2 (version: 3.2.1) and pheatmap (version: 1.0.12), respectively. The Spearman correlation test between DEGs and abundance of TIICs were conducted, and the immune-related DEGs were selected with the threshold of P value < 0.05 and |r| > 0.15.

### Functional enrichment analysis

To investigate the functional involvement of the DEGs, clusterProfiler 23 (version: 3.12.0) in R package was used to conduct the gene ontology (GO) terms and KEGG pathways enrichment analysis for all the DEGs based on Fisher exact test. GO terms included three categories: biological process (BP), cellular component (CC), and molecular function (MF). The significantly enriched GO terms and KEGG pathways were selected with the cut-off of P value < 0.05.

### Protein-protein interaction (PPI) network

The interactions between DEGs were retrieved from STRING (version: 10.0, http://string-db.org/) database with a minimum required interaction score setting of 0.900 (highest confidence). Then, the PPI network was visualized using Cytoscape software, in which the functional modules were identified using MCODE plugin 24 of Cytoscape. The functional modules with score > 10 were selected, and the immune related DEGs in the functional modules were marked.

### Survival analysis of DEGs

The prognosis-related clinical data including overall survival (OS) and smoking history were retrieved. The samples were divided into high-expression and low-expression groups based on the median value of each gene expression, together with log-rank statistical test. The significant threshold was set as P value < 0.05 to select the genes associated with prognosis, and then Kaplan-Meier (K-M) survival curves were plotted.

## Results

### Basic characteristics of samples

Detailed clinical and pathological characteristics of the 519 LUAD patients and 550 LUSC patients are shown in Table 1A and Table 1B, respectively. The 519 LUAD patients smoked 41.32 ± 26.93 years with OS of 31.86 ± 30.58 months, of which 184 patients (35.45%) died. The 550 LUSC patients smoked 52.66 ± 31.08 years with OS of 36.90 ± 38.09 months, of which 286 patients (52.00%) died.

A total of 496 LUAD and 490 LUSC samples had data pertaining to both gene expression and clinical phenotype. A total of 19,712 genes were obtained in both LUAD and LUSC samples after re-annotation, and finally 17,522 and 17,751 genes were obtained in the LUAD and LUSC samples, following filtering out of genes with low expression values.

### Immune score and immune cytolytic activity score in LUAD and LUSC

**Figure 1:**
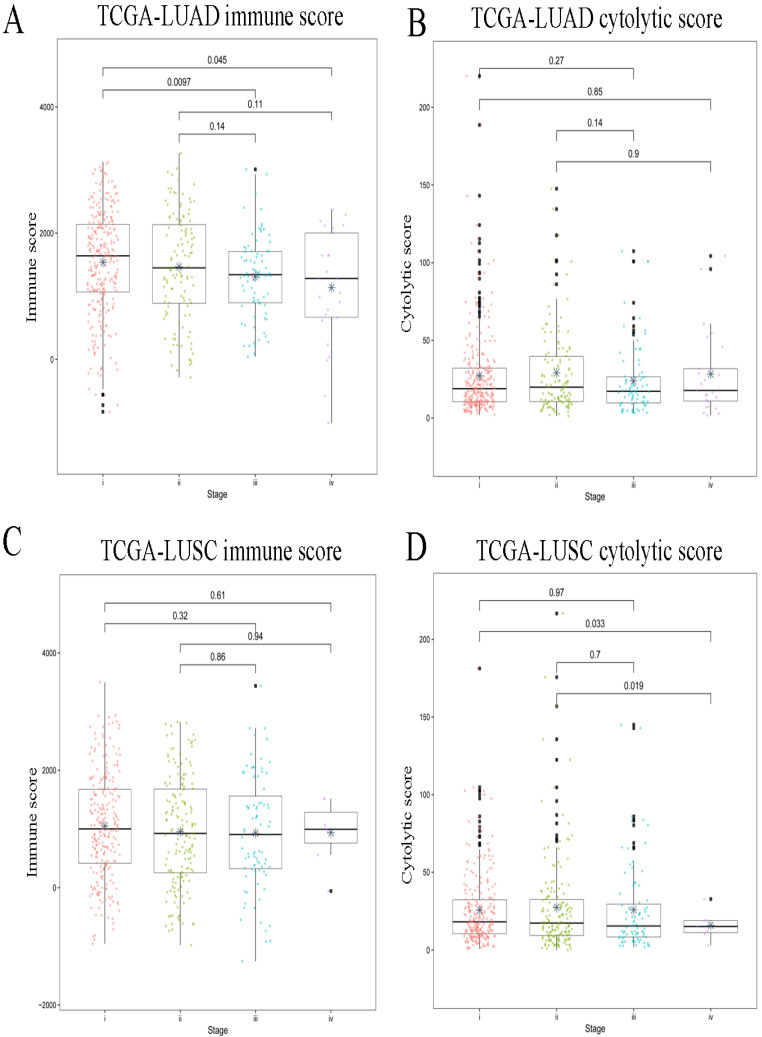
** Immune scores and immune cytolytic activity scores between early and advanced stages in LUAD and LUSC. (A)** Distribution of immune scores in LUAD. **(B)** Distribution of immune cytolytic activity scores in LUAD.** (C)** Distribution of immune scores in LUSC. **(D)** Distribution of immune cytolytic activity scores in LUSC. The horizontal line represents the median value; asterisks represent the mean value. The number represents P value, and P < 0.05 shows significant statistical difference between two stages.

The immune cells from the tumor immune microenvironment play an important role in tumor progression. The density and location of such immune cells can be quantified by an immune score which is considered to be a tangible indicator of prognosis of the tumor. The cytolytic activity score has been reported to be related to improved prognosis and anti-regulatory activities that limit the immune response 17. In this study, for the LUAD subtype, there was significant difference in the immune scores between the early and advanced stages, and stage I showed the highest immune score out of the four stages (Figure 1A). Further, the immune cytolytic activity score among the stages was also calculated, however, no significant difference was found among the stages (Figure 1B). In the LUSC subtype, there was no significant difference in the immune score between the early and advanced stages (Figure 1C). Conversely, the distribution of immune cytolytic activity score was different from the immune score. The early stages showed higher immune cytolytic activity score than the advanced stage (Figure 1D).

### TMB and neoantigens in LUAD and LUSC

TMB is considered as a promising indicator to predict the effect of immune checkpoint inhibitors, and it is associated with the neoantigen burden 25. Chen et al. demonstrated that high TMB and neoantigen burden was significantly correlated with improved immunotherapeutic effect in NSCLC 26. Therefore, TMB and neoantigens were analyzed for all the samples and the different stages in LUAD and LUSC.

**Figure 2:**
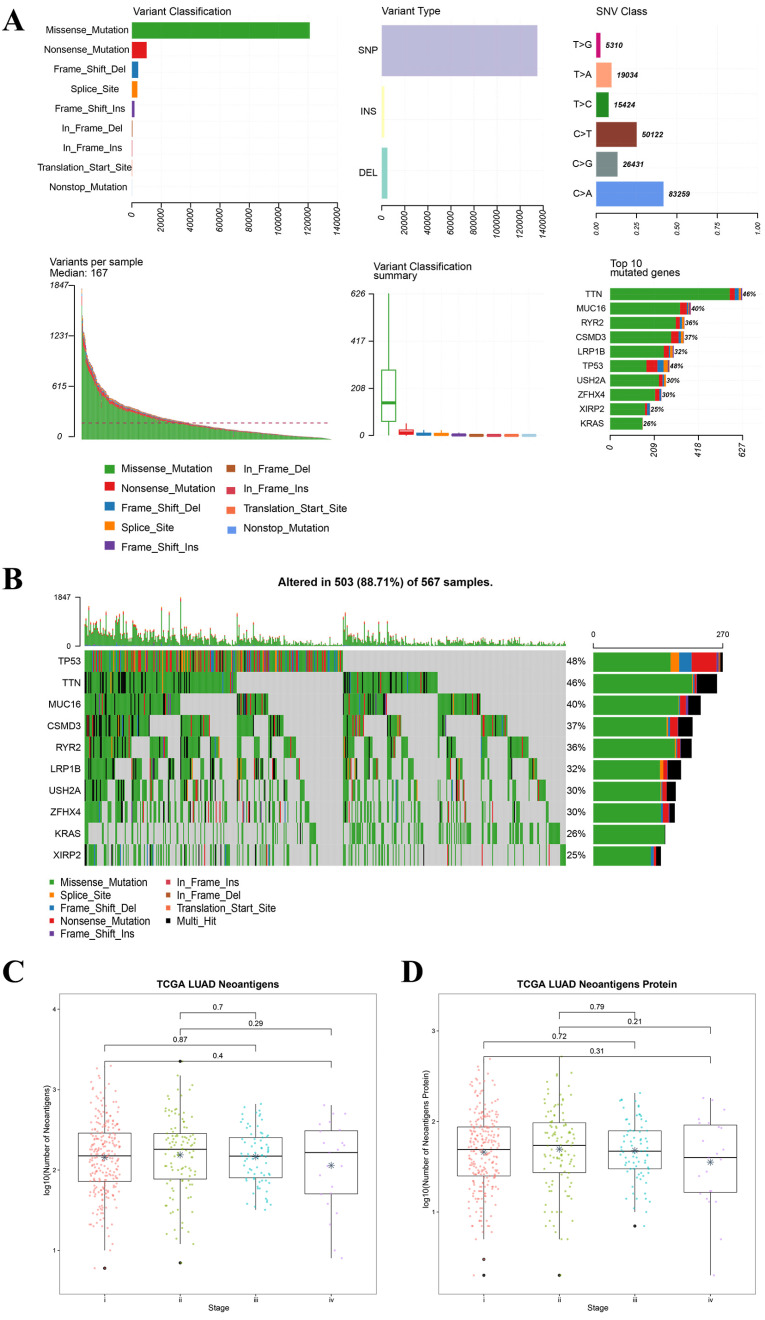
** Landscape of TMB and neoantigens in LUAD. (A)** The summary plot of TMB in LUAD. **(B)** The oncoplot of TMB in LUAD. **(C)** Distribution of neoantigen burden in LUAD. **(D)** Distribution of neoantigen origin protein in LUAD. Missense mutation account for most of the variant classification, followed by nonsense mutation; single nucleotide polymorphisin (SNP) is the major variant type; C>A is the major site of single nucleotide variation (SNV), followed by C>T; median of variants per sample is 167; List of top 10 mutated genes in summary plot shows the top 10 genes ordered by total number of variants in each gene, and the percentage following each gene represent the ratio of samples with this gene variation to total samples. Oncoplot shows the list of top 10 gene ordered by the the number of samples with the gene variants, and the percentage represent the ratio of samples with this gene variation to total samples. The horizontal line and asterisks in panel C and D represent median value and mean value, respectively. The number represents P value, and P < 0.05 shows significant statistical difference between two stages

**Figure 3:**
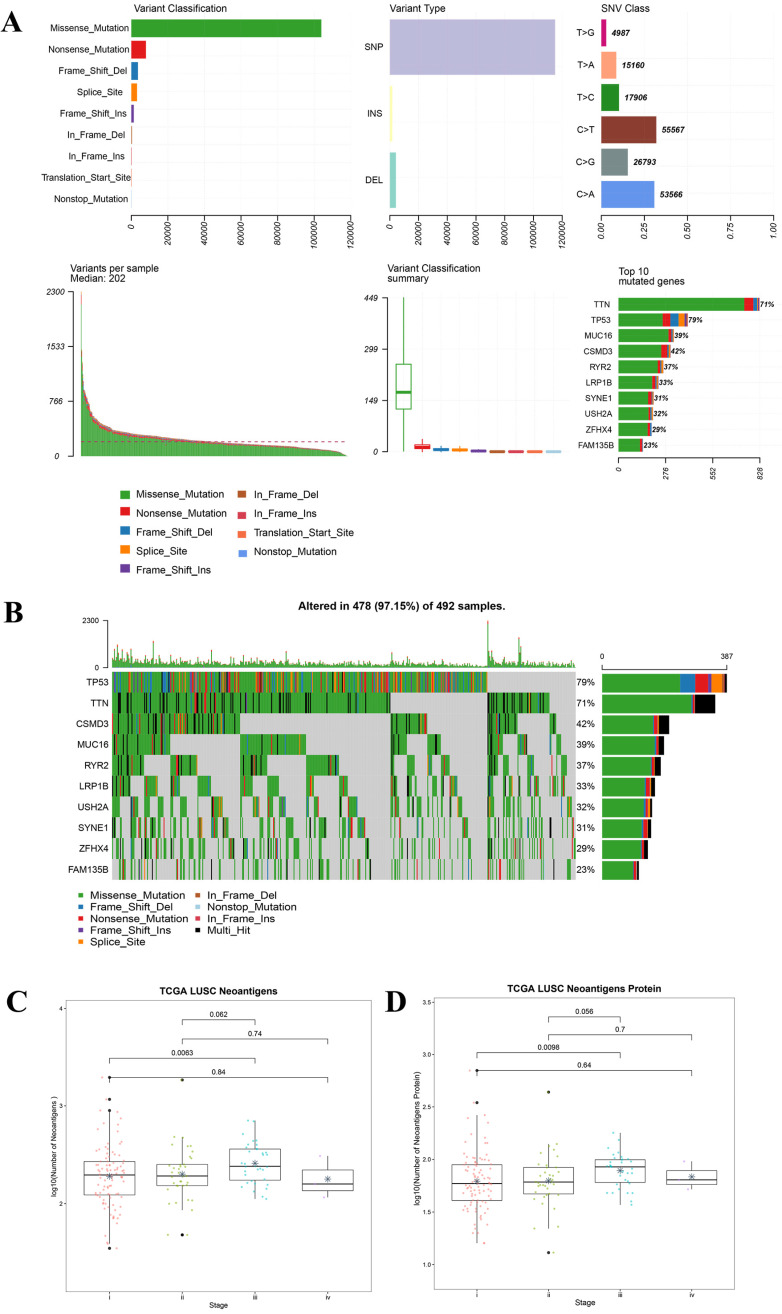
** Landscape of TMB and neoantigens in LUSC. (A)** The summary plot of TMB in LUSC. **(B)** The oncoplot of TMB in LUSC. **(C)** Distribution of neoantigen burden in LUSC. **(D)** Distribution of neoantigen origin protein in LUSC. Missense mutation account for most of the variant classification, followed by nonsense mutation; single nucleotide polymorphisin (SNP) is the major variant type; C>T is the major site of single nucleotide variation (SNV), followed by C>A; median of variants per sample is 202; List of top 10 mutated genes in summary plot shows the top 10 genes ordered by total number of variants in each gene, and the percentage following each gene represent the ratio of samples with this gene variation to total samples. Oncoplot shows the list of top 10 gene ordered by the the number of samples with the gene variants, and the percentage represent the ratio of samples with this gene variation to total samples. The horizontal line and asterisks in panel C and D represent median value and mean value, respectively. The horizontal line and asterisks in panel C and D represent median value and mean value, respectively. The number represents P value, and P < 0.05 shows significant statistical difference between two stages.

For all the LUAD samples, the most frequent variant was missense mutation, followed by nonsense mutation. Single nucleotide polymorphism (SNP) was responsible for such variants, and single nucleotide variants (SNVs) mostly occurred as C > A and C > T (Figure 2A). Similar results were found in samples of different tumor stages (data not shown). The top 10 mutated genes in all the LUAD samples were TP53, KRAS, XIRP2, ZFHX4, USH2A, LRP1B, CSMD3, RYR2, MUC16, and TTN (Figure 2B), and these are listed in Table 2A. Similarly, for all the LUSC samples and samples with different tumor stages, the most frequent variant was missense mutation, followed by nonsense mutation. SNP was responsible for such variants, and the SNV mostly occurred as C > A and C > T (Figure 3A). The top 10 mutated genes in all the LUSC samples were TP53, FAM135B, ZFHX4, USH2A, SYNE1, LRP1B, RYR2, CSMD3, MUC16, and TTN (Figure 3B), and these are listed in Table 2B. The top 10 overlapping mutated genes were TP53, ZFHX4, USH2A, LRP1B, CSMD3, RYR2, MUC16 and TTN. The mutated genes KRAS and XIRP2 were identified only in LUAD, while FAM135B and SYNE1 were identified only in LUSC. Investigation of the neoantigens and neoantigen origin proteins revealed that there were no significant differences in the neoantigen burden in early vs. advanced stages in LUAD (Figure 2C-D). However, in LUSC, stage III had significantly higher neoantigen burden compared to stage I (Figure 3C-D).

### Immune cells infiltration in LUAD and LUSC

**Figure 4:**
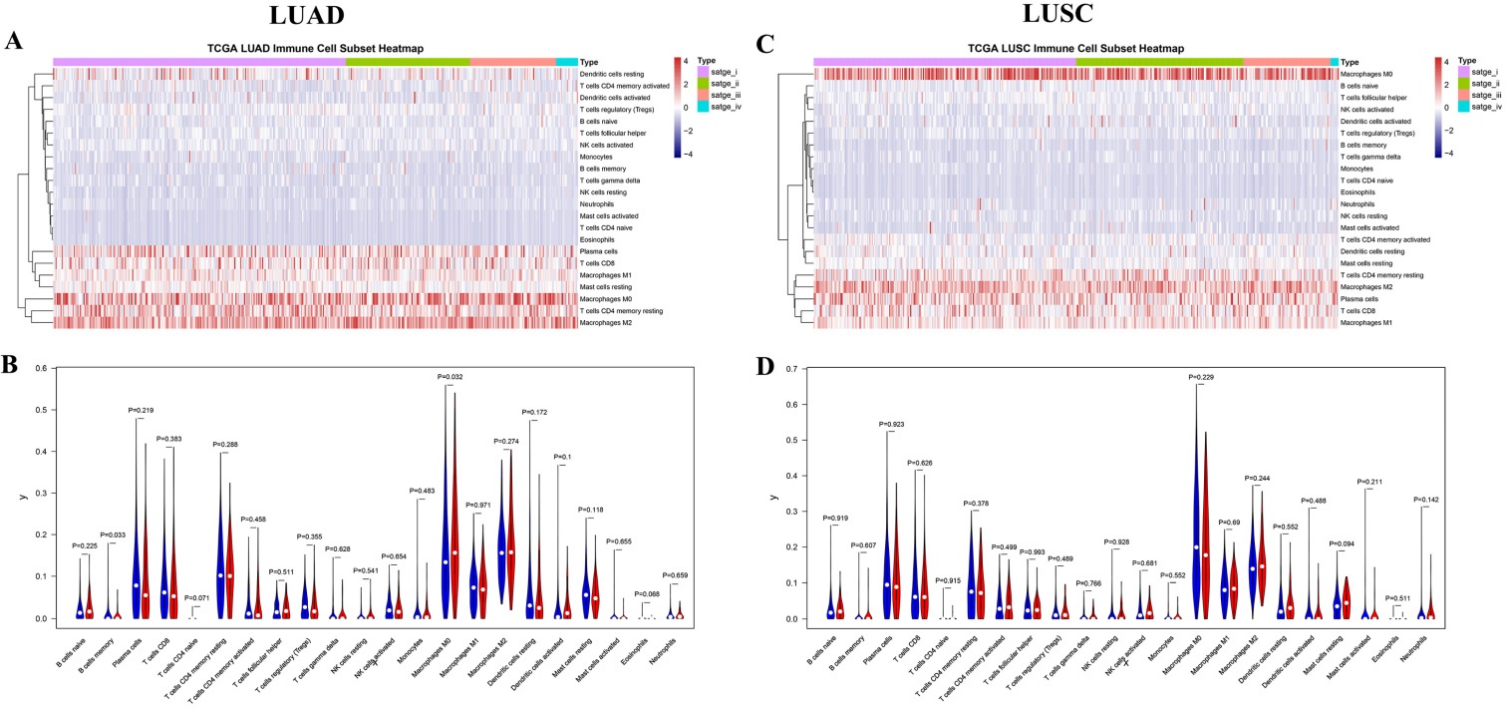
** The landscape of immune infiltration in LUAD and LUSC. (A)** Immune cell subset heatmap in LUAD. **(B)** Violin plot of early and advanced stage in LUAD. **(C)** Immune cell subset heatmap in LUSC. **(D)** Violin plot of early and advanced stage in LUSC. The dots in Violin plot represent the median value, and the blue color and red color represent early stage and advanced stage, respectively. P < 0.05 shows significant statistical difference between two stages.

The 22 different immune cell types among different tumor stages in LUAD and LUSC subtypes were analyzed using the CIBERSORT algorithm. The bar charts of immune cell subset proportions (Supplemental Figure S1) and immune cell subset heatmaps (Figure 4A) revealed that the M0 macrophages, M2 macrophages, and resting memory CD4 T cells accounted for a large proportion of immune cell infiltration in LUAD. Further, the differences in the proportion of TIICs in early (stages I and II) vs. advanced stages (stages III and IV) were also investigated. Early stage displayed greater number of memory B cells and M0 macrophages compared to the advanced stage in LUAD (Figure 4B). In addition, the associations between OS and TIIC abundance, and between tumor stage and TIIC abundance, were also investigated (Table 3). We found that the abundance of M0 macrophage infiltration was significantly correlated with both the tumor stage (P = 0.016) and OS (P = 0.049) (Supplemental Figure S2).

Similarly, M0 macrophages, M2 macrophages, and plasma cells accounted for a large proportion of the immune cell infiltration in LUSC (Figure 4C, Supplemental Figure S3). However, no significant difference was observed in the proportion of TIICs in the early vs. advanced stages in LUSC (Figure 4D). Although, the abundance of resting (P = 0.042) and activated mast cells (P = 0.032) were found to be significantly correlated with the tumor stages. Four immune cell types including resting dendritic cells, eosinophils, activated memory CD4 T cells, and mast cells were significantly correlated with OS (Table 3, Supplemental Figure S4).

### Screening and functional enrichment of DEGs

**Figure 5:**
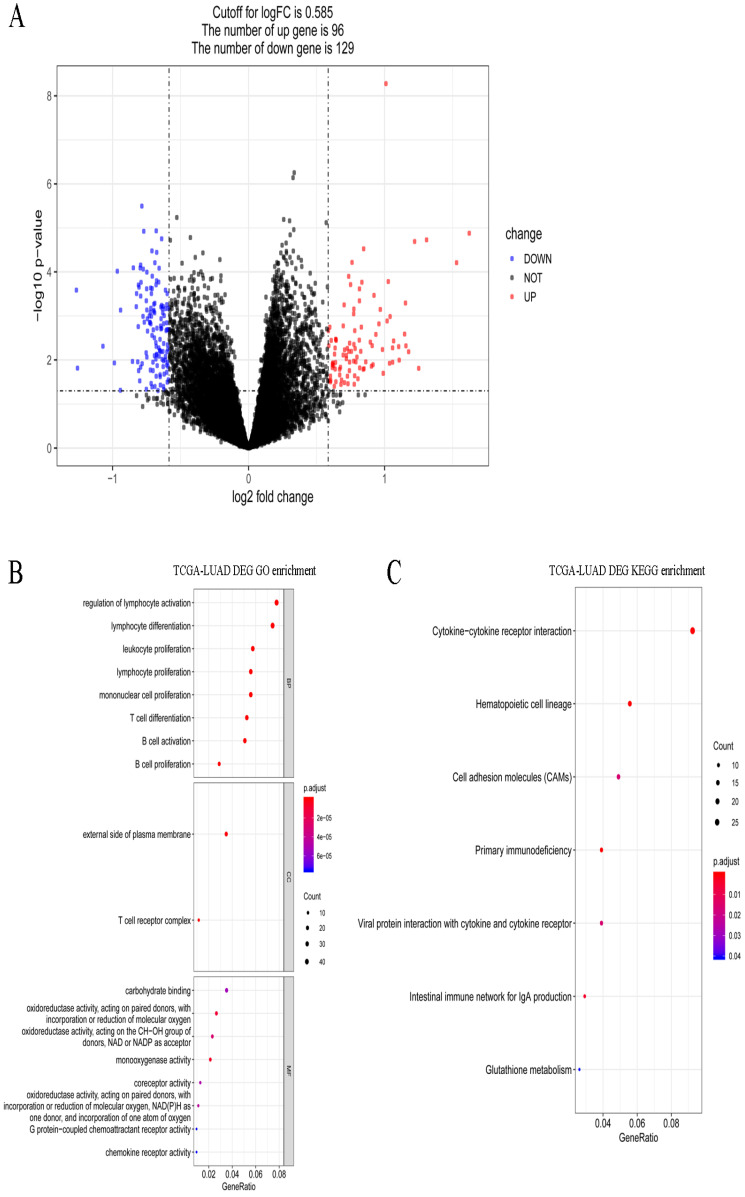
** Gene expression spectrum and functional enrichment in LUAD. (A)** The volcano plots of DEGs between stages I and IV in LUAD. Red dots and blue dots represent the significant up-regulated and down-regulated DEGs, respectively. **(B)** The significantly enriched GO terms for DEGs between stages I and IV in LUAD. **(C)** The significantly enriched KEGG pathways for DEGs between stages I and IV in LUAD.

**Figure 6:**
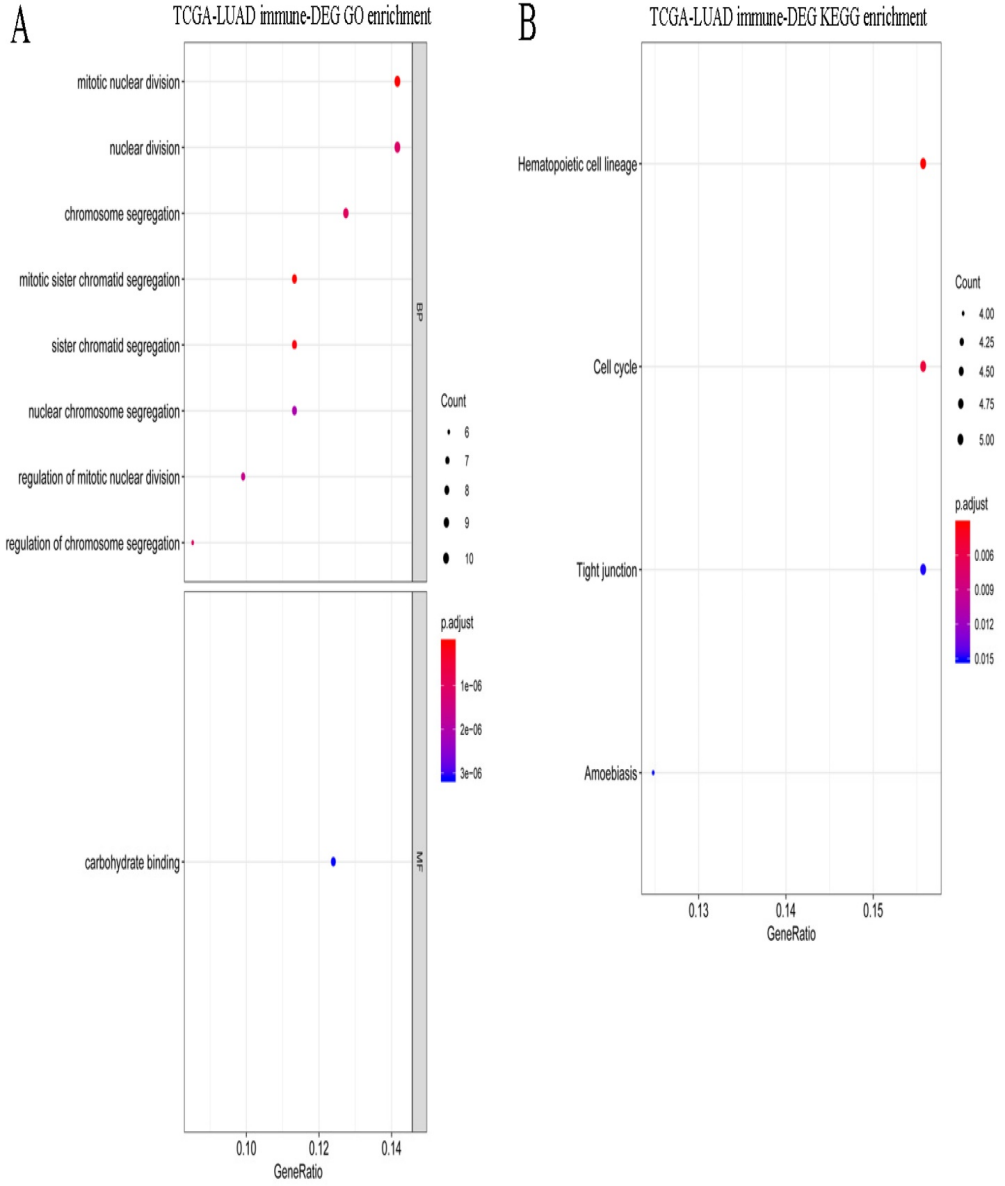
** Functional enrichment for immune-related DEGs in LUAD. (A)** The significantly enriched GO terms for immune-related DEGs in LUAD. **(B)** The significantly enriched KEGG pathways for immune-related DEGs in LUAD.

A total of 495 DEGs were screened between stages I and IV in LUAD, including 232 upregulated and 263 downregulated genes. Volcano plots of the DEGs are shown in Figure 5A. These DEGs were significantly enriched in the various GO terms and KEGG pathways, such as regulation of lymphocyte activation, leukocyte proliferation, and hematopoietic cell lineage (Figure 5B-C). Then, the DEGs that correlated with the abundance of M0 macrophage infiltration were identified using Spearman correlation test. A total of 83 immune-related DEGs were identified in LUAD which were significantly enriched in mitosis-related biological processes, such as mitotic nuclear division, mitotic sister chromatid segregation, and nuclear division, and enriched in several KEGG pathways, such as hematopoietic cell lineage, cell cycle, and tight junction (Figure 6A-B).

**Figure 7:**
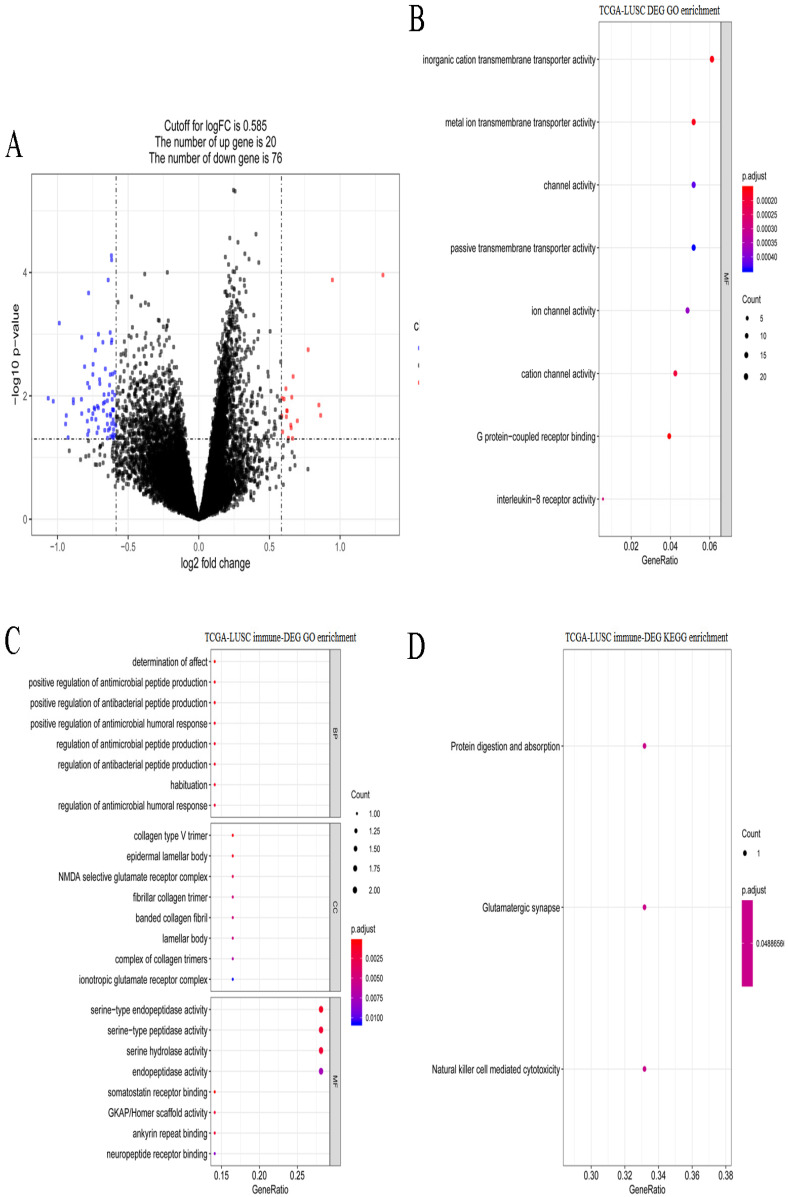
** Gene expression spectrum and functional enrichment in LUSC. (A)** The volcano plots of DEGs between stages I and IV in LUSC. Red dots and blue dots represent the significant up-regulated and down-regulated DEGs, respectively. **(B)** The significantly enriched GO terms for DEGs between stages I and IV in LUSC. **(C)** The significantly enriched GO terms for immune-related DEGs in LUSC. **(D)** The significantly enriched KEGG pathways for immune-related DEGs in LUSC.

A total of 621 DEGs (492 upregulated and 129 downregulated) were screened between stages I and IV in LUSC (Figure 7A). These DEGs were significantly enriched 8 molecular function terms (Figure 7B), including, G protein-coupled receptor binding. None of the KEGG pathways were enriched. A total of 9 immune-related DEGs were identified in LUSC, and they were enriched in antibacterial-related biological processes and 3 KEGG pathways, including protein digestion and absorption, glutamatergic synapse, and natural killer cell mediated cytotoxicity (Figure 7C-D).

### PPI network and functional modules

**Figure 8:**
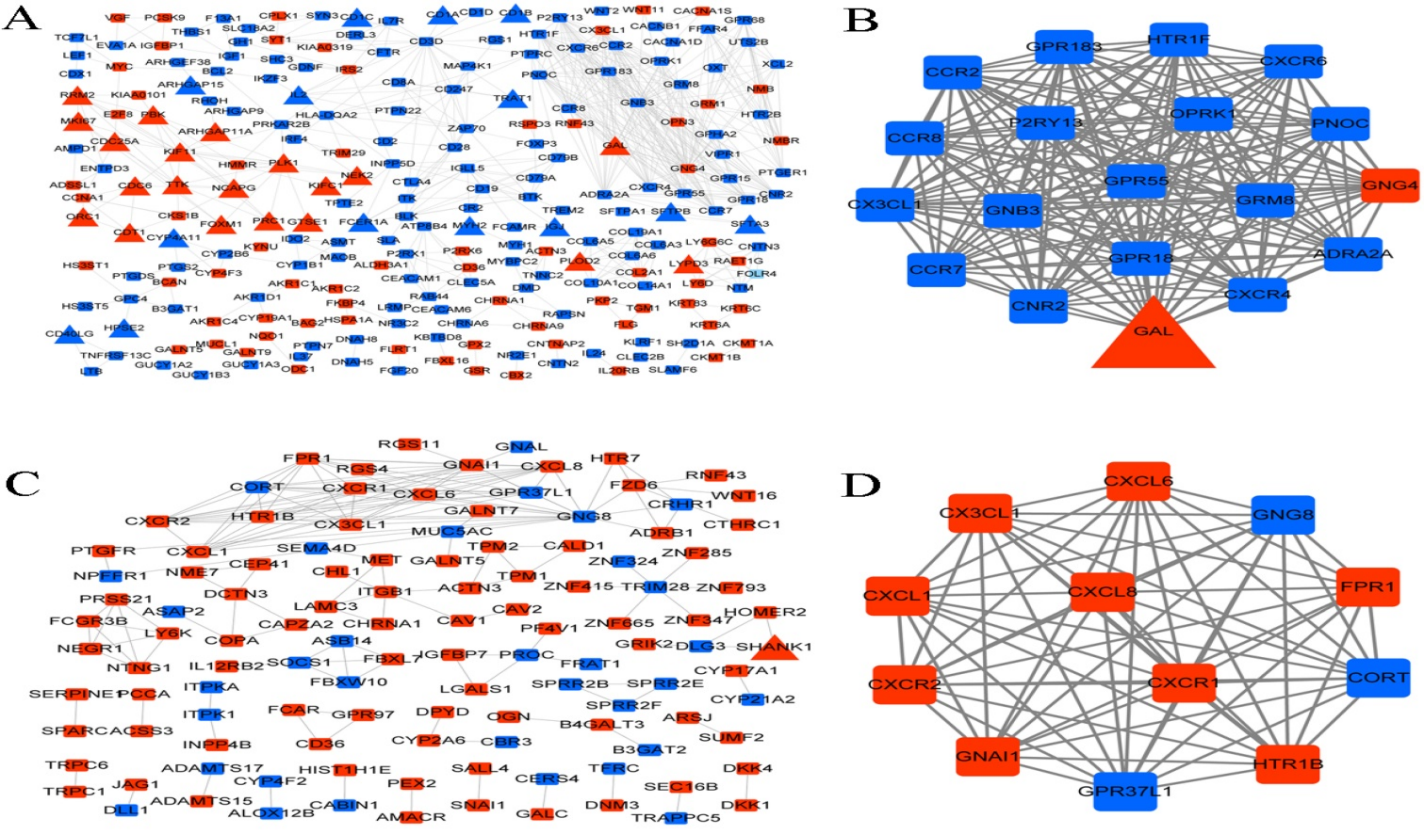
** PPI network and functional modules. (A)** PPI network of DEGs in LUAD. **(B)** The identified functional module with score > 10 in LUAD. **(C)** PPI network of DEGs in LUSC. **(D)** The identified functional module with score > 10 in LUSC. Square and triangle represent the DEGs and immune-related DEGs, respectively. Red nodes and blue nodes represent the significant up-regulated and down-regulated genes, respectively.

Proteins and their functional interactions form the backbone of the cellular machinery, and their connectivity networks will futher the understanding of biological phenomena 27. Therefore, PPI networks were constructed based on the interactions in STRING database. In LUAD, the PPI network contained 233 genes, of which 35 were immune-related genes, and one module with score > 10 was identified from the PPI network (Figure 8A-B). The module contained one immune-related gene (GAL), one upregulated gene (GNG4), and 17 downregulated genes.

In LUSC, the PPI network contained 116 genes, of which only one was an immune-related gene (SHANK1), and one module with score > 10 was identified from the PPI network (Figure 8C-D). The module contained 12 genes, including 3 downregulated and 9 upregulated genes.

### Survival analysis

Survival analysis was performed for the identified immune-related DEGs. Among the 83 immune-related DEGs in LUAD, 23 genes were found to have significant correlation with survival. Only two genes, including SHANK1 and COL5A3, were significantly correlated with survival among the 9 immune-related DEGs in LUSC (Table 4).

## Discussion

In the current study, the gene expression and clinical phenotype data of LUAD and LUSC samples were used to analyze the immune microenvironment among different histological types and clinical stages. We found that there were some similarities and differences in the immune microenvironment of LUAD and LUSC. In the LUAD subtype, the early stage had a higher immune score than the advanced stage, but there was no significant difference in the immune cytolytic activity score or neoantigen burden. While in LUSC, the early stage was with a higher immune cytolytic activity score and neoantigen burden than the advanced stage, but there was no significant difference in the immune score. Roufas et al. demonstrated that the levels of immune cytolytic activity varied greatly in different cancer types, and the cytolytic index along with complex associations among different TIICs was able to promote evasion from immune surveillance 28.

Our results indicated that TIICs and cell types were significantly correlated with progression and survival, of which the abundance of macrophage infiltration was associated with clinical stages and prognosis in LUAD, while the abundance of mast cells infiltration was associated with clinical stages in LUSC. Mast cells are well known for their roles in allergic disorders 29. Recently, mast cells were found to play an important role in controlling inflammatory responses. They could not only produce a variety of inflammatory mediators, but also ameliorate inflammation by producing immune regulatory factors (IL-10) 30. Mast cell infiltration has been implicated in metastasis and angiogenesis in several human malignancies 31. Tryptase mast cell infiltration was found to greatly reduce the OS and disease-free survival in various solid tumors, and was significantly related to worse OS in NSCLC 32. Mast cells infiltration and its number in the tumor microenvironment in lung cancer was also associated with poor prognosis 33. Zhang et al. demonstrated that tumor infiltration by macrophages was associated with tumor lymph angiogenesis and unfavorable prognosis in LUAD 34. Kawai et al. showed that macrophages infiltration could indicate prognosis of patients with stage IV NSCLC 35. The results indicated that mast cell and macrophages infiltration in the tumor microenvironment was associated with prognosis. The densities of mast cells and macrophages should be evaluated by immunohistochemical staining in clinical samples, and their relationships with clinicopathological factors and prognosis should be further investigated.

TMB has been considered as a useful biomarker for response to immune therapy and prognosis in lung cancer 36. In TMB analysis, the median of variants per sample showed a decrease from stage I to IV in LUAD, while it increased from stage I to IV in LUSC. Of the top 10 mutated genes KRAS and XIRP2 were only present in LUAD, while FAM135B and SYNE1 were only present in LUSC. However, in both LUAD and LUSC, the most frequent variant was missense mutation, followed by nonsense mutation. SNP was responsible for such variants, and the SNV mostly occurred as C > A and C > T. The main mutated genes were TP53, ZFHX4, USH2A, LRP1B, CSMD3, RYR2, MUC16, and TTN. Owada-Ozaki et al. reported that high TMB in post-operation NSCLC patients might indicate unfavorable prognosis, and stage I NSCLC patients with high TMB showed higher recurrence rate 37. While Devarakonda et al. showed that high TMB could be a better prognosis for patients with resected NSCLC 38. Such differences in the two studies might be associated with histologic subtypes. The distribution of TMB and the subsets of patients with high TMB had not been well described in most cancer types. Based on the analysis of human cancer genomes, Chalmers et al. found the median mutations for LUAD was 6.3 Mb and for LUSC was 9.0 Mb, and suggested that many cancer types have a substantial portion of patients with high TMB who might benefit from immunotherapy 39. For the clinical application of TMB, validated assays and proper thresholds are indispensable. To make TMB function as a more reliable predictive biomarker, there were requirements for extra biologic guidance or computational assistance to predict associated-neoantigens 40. It was suggested that highly mutated tumors were more likely to harbor neoantigens which make them targets of activated immune cells. Neoantigen is bound to the major histocompatibility complex (MHC) molecules and exists on the surface of tumor cells in the form of a protein complex. It can be specifically recognized by the cytotoxic T cell receptor (TCR) to activate the immune response of T cells. Studies had reported that tumor neoantigen plays a pivotal role in immune escape, anti-tumor immune response and immunotherapy 41-43.

Further, a total of 495 and 621 DEGs were identified between stages I and IV in LUAD and LUSC, respectively. The DEGs in LUAD were significantly enriched in T lymphocyte cell differentiation, etc. While the DEGs in LUSC were mainly enriched in metal ion transmembrane transporter activity, G protein-coupled receptor binding, etc. This suggested that the DEGs in LUAD and LUSC were different, and could be used to distinguish two subtypes. Of these, 83 immune-related DEGs were identified in LUAD of which 23 genes (including, PLK1 and RRM2) were found to be correlated with survival, while 9 immune-related DEGs were identified in LUSC and 2 (including, SHANK1) were found to be correlated with survival. PLK1, Polo Like Kinase 1, encodes a Ser/Thr protein kinase of the CDC5/Polo subfamily that is essential in mitotic progression 44. Jolien et al. demonstrated that the level of PLK1 was increased in LUAD, and the combined evaluation of PLK1, carbonic anhydrase IX, and TP53 could predict prognosis of LUAD patients 45. Noboru et al. showed that the expression of karyopherin beta 1 could be decreased by inhibiting PLK1, and such a decrease could inhibit cell proliferation via apoptosis in LUAD cells 46. Ribonucleotide reductase (RR) plays a crucial role in DNA replication and repair. It consists of RRM1 and RRM2 subunits, and its enzymatic activity is regulated mainly by RRM2 subunit 47. RRM2 has been reported to have an active role in the progression of multiple cancers, including NSCLC 48. Previous studies have reported that low expression of RRM1 and RRM2 could increase the response of NSCLC patients undergoing platinum-based chemotherapy, and could indicate a better outcome in NSCLC 49, 50. RRM2, but not RRM1, could be a valuable biomarker to predict survival outcome of women, non-smokers, and former smokers who had stopped smoking for at least 10 years 51. SHANK1, SH3 and multiple ankyrin repeat domains 1, a member of the SHANK family, are scaffold proteins that function in the establishment of dendritic spines 52. SHANK1 is found to be expressed at high levels in lung cancer tissues compared to para cancerous tissues, however, its high expression had no significant correlation with gender, age, pathological grade or classification except with T stage 53. In our study, PLK1 and RRM2 were identified as immune-related DEGs, and were found to be significantly associated with survival in LUAD. SHANK1 was the only immune-related DEG in the PPI network in LUSC, and it was significantly associated with survival in LUSC. Hence, we concluded that PLK1 and RRM2 may be useful biomarkers to predict survival outcome in LUAD, and SHANK1 was a crucial biomarker in LUSC. Further clinical study, both in vitro and in vivo experiments are necessary to validate the expressions of PLK1, RRM2, and SHANK1 and their roles in proliferation, metastasis and invasion. In addition, further clinical studies are required to determine whether those genes were independent prognosis biomarkers as well as its associations with immunotherapy efficacy.

Despite of the several novel findings, there still remained some limitations. (1) Multiple hypothesis test in statistical analyses should be taken into account. Moreover, TCGA clinical data for staging include different AJCC staging editions. (2) Tumor immune microenvironment characteristics, including immune score, immune cytolytic activity score, neoantigen burden, were analyzed. The clinical application in immunotherapy of these characteristics should be further investigated with large sample size. (3) Small cell lung cancer (SCLC) accounts for approximately 15% of lung cancer, however the study of immune microenvironment characteristics and involved key genes were still limited and largely unknown. Further studies in SCLC are still needed.

In conclusion, the characteristics of immune microenvironment and genes involved in the different stages of LUAD and LUSC were investigated. These findings may provide theoretical basis in further studies and in accurate personalized immunotherapy for NSCLC patients.

## Supplementary Material

Supplementary figures and tables.

## Figures and Tables

**Table 1:** The clinical and pathological characteristics of 519 LUAD patients and 550 LUSC patients

Clinical	Describe
**(A) Basic characteristics of LUAD samples**	
pathologic_M (M1/M0)	26/346
pathologic_N (N3/N2/N1/N0)	32/72/96/335
pathologic_T (T4/T3/T2/T1)	17/45/278/174
Stage (IV /III / II / I)	27/81/124/287
Group (advance/ early)	108/411
OS (months)	31.86±30.58
Survival_status (Dead/Alive)	184/335
Smoked years	41.32±26.93
**(B) Basic characteristics of LUSC samples**	
pathologic_M (M1/M0)	7/407
pathologic_N (N3/N2/N1/N0)	23/46/131/293
pathologic_T (T4/T3/T2/T1)	23/69/292/115
Stage (IV /III / II / I)	7/86/161/245
Group (advance / early)	93/406
OS (months)	36.90±38.09
Survival_status (Dead/Alive)	213/286
Smoked years	52.66±31.08

**Table 2:** The top 10 mutated genes in samples with different tumor stages

(A) The top 10 mutated genes in LUAD samples with different tumor stages							
Stage I	Proportion	Stage II	Proportion	Stage III	Proportion	Stage IV	Proportion
TP53	46%	TTN	50%	TTN	50%	TP53	56%
TTN	43%	TP53	50%	TP53	46%	KEAP1	44%
MUC16	41%	CSMD3	41%	MUC16	41%	CSMD3	40%
CSMD3	38%	RYR2	38%	RYR2	36%	SPTA1	40%
RYR2	35%	MUC16	37%	LRP1B	35%	TTN	32%
LRP1B	33%	USH2A	34%	USH2A	30%	RYR2	28%
ZFHX4	32%	KRAS	31%	KRAS	28%	RYR3	28%
USH2A	31%	ZFHX4	31%	CSMD3	25%	KRAS	24%
KRAS	25%	LRP1B	30%	FLG	24%	RP1L1	24%
FLG	25%	SPTA1	30%	COL11A1	22%	ZFHX4	24%
(B) The top 10 mutated genes in LUSC samples with different tumor stages							
Stage I	Proportion	Stage II	Proportion	Stage III	Proportion	Stage IV	Proportion
TP53	81%	TP53	78%	TP53	73%	MUC16	86%
TTN	71%	TTN	71%	TTN	71%	TP53	86%
CSMD3	39%	CSMD3	44%	CSMD3	49%	TTN	86%
MUC16	38%	MUC16	38%	RYR2	43%	ADGRL3	71%
RYR2	36%	RYR2	34%	MUC16	40%	CUBN	71%
LRP1B	34%	SYNE1	32%	USH2A	38%	ZFHX4	71%
USH2A	31%	USH2A	31%	LRP1B	33%	CSMD3	57%
SYNE1	30%	LRP1B	31%	SYNE1	29%	LRP1B	57%
ZFHX4	28%	ZFHX4	30%	NAV3	28%	PCDH15	57%
KMT2D	23%	CDH10	23%	XIRP2	27%	SYNE1	57%

**Table 3:** Analysis of the abundance of tumor infiltrating immune cells

	LUAD		LUSC	
	Immune cell types	P value	Immune cell types	P value
Stages	Macrophages M0	0.016	Mast cells resting	0.042
	/		Mast cells activated	0.032
Overall survival	Macrophages M0	0.049	Dendritic cells resting	0.024
	/	/	Eosinophils	0.017
	/	/	T cells CD4 memory activated	0.047
Immune cell clustering	Macrophages M0	/	Macrophages M0	/
	T cells CD4 memory resting	/	Macrophages M2	/
	Macrophages M2	/	Plasma cells	/
Stage I vs III	B cells memory	0.015	/	/
	Macrophages M0	0.04	/	/
	Mast cells resting	0.011	/	/
	Eosinophils	0.043	/	/
Stage I vs IV	Mast cells activated	0.015	B cells memory	0.001
Stage II vs III	/	/	Mast cells resting	0.01
	/	/	Mast cells activated	0.033
Stage II vs IV	/	/	B cells memory	0.006

**Table 4:** Immune-related DEGs correlated with survival

LUAD		LUSC	
Gene name	P value	Gene name	P value
LYPD3	0.001	SHANK1	0.007
PLK1	0.002	COL5A3	0.009
RRM2	0.002		
PLOD2	0.003		
KIF11	0.004		
EXO1	0.006		
SPOCK1	0.006		
PTPRN	0.007		
GTSE1	0.008		
PRC1	0.009		
CDC6	0.010		
EGLN3	0.013		
FAM72D	0.014		
FOXM1	0.014		
CYP4A11	0.018		
FAM64A	0.018		
NEK2	0.021		
CDC25A	0.024		
TTK	0.028		
ARHGAP11A	0.029		
CA10	0.031		
ORC1	0.041		
NCAPG	0.046		
