# Supplementary material for: Identification of the key genes and characterizations of Tumor Immune Microenvironment in Lung Adenocarcinoma (LUAD) and Lung Squamous Cell Carcinoma (LUSC)
Source: J Cancer. 2020 Jun 16;11(17):4965–79. doi: 10.7150/jca.42531 (PMC7378909; doi:10.7150/jca.42531)
Supplement: Supplementary file 1 — Supplementary figures and tables. [file jcav11p4965s1.pdf]

Supplemental Figure 1. The bar charts of immune cell subset proportions in LUAD.

The bar charts of immune cell subset proportions in stage I (A), stage II (B), stage III (C), and stage IV (D). The horizontal axis represent samples, and the vertical axis represent the percent of immune cells infiltration.

Supplemental Figure 2. Associations between tumor stages/survival and the abundance of TIICs in LUAD. (A) The boxplot of M0 macrophage infiltration in different stages. The horizontal line represents the median value; (B) Survival curve of abundance of M0 macrophage infiltration in LUAD.

Supplemental Figure 3. The bar charts of immune cell subset proportions in LUSC.

The bar charts of immune cell subset proportions in stage I (A), stage II (B), stage III (C), and stage IV (D). The horizontal axis represent samples, and the vertical axis represent the percent of immune cells infiltration.

Supplemental Figure 4. Associations between tumor stages/survival and the abundance of TIICs in LUSC. The boxplot of activated mast cells infiltration (A) and resting mast cells infiltration (B) in different stages. The horizontal line represents the median value; Survival curves of infiltration abundance of resting dendritic cell, eosinophils, and activated memory CD4 T cells in LUSC.

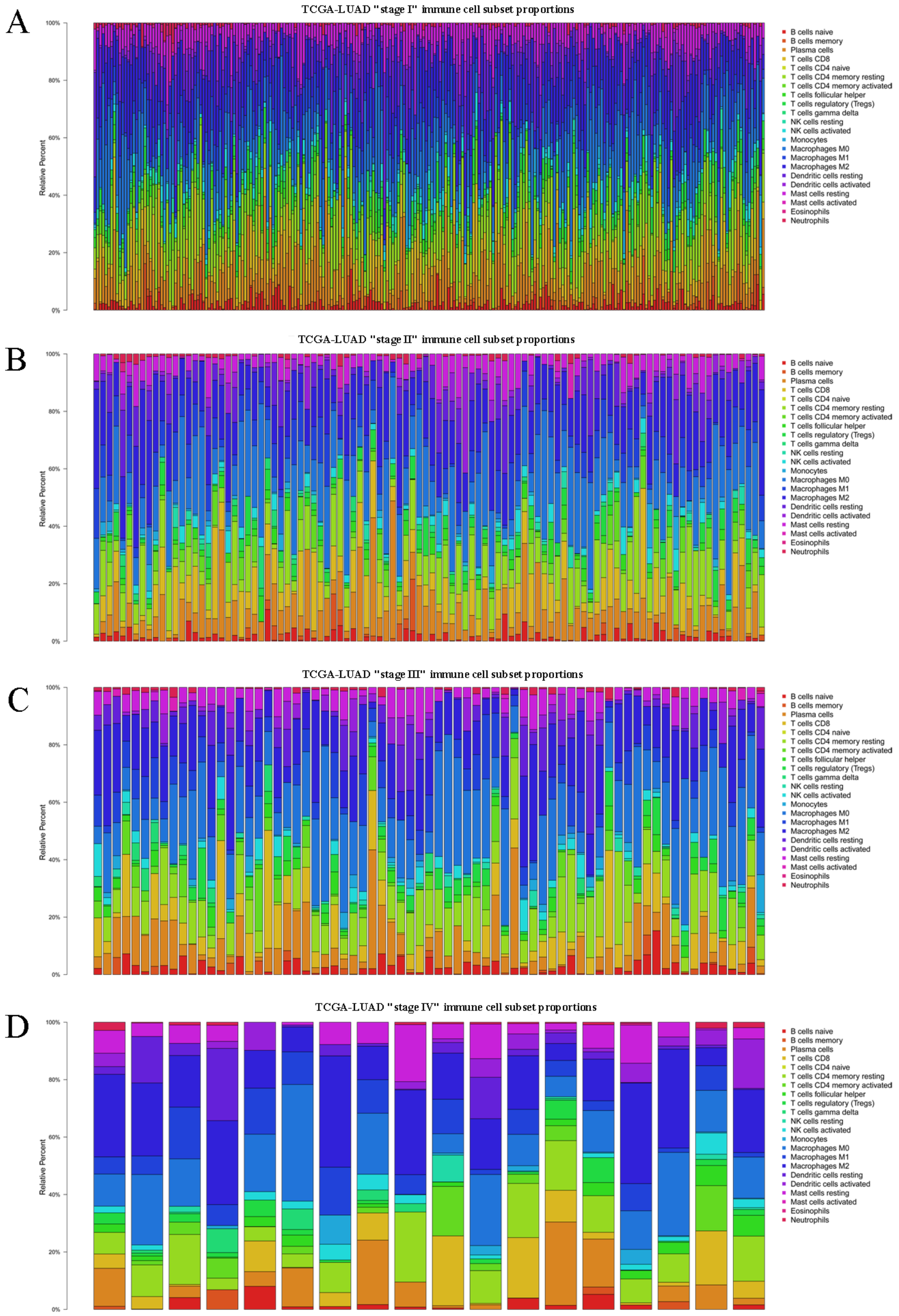

Supplemental Figure 1. The bar charts of immune cell subset proportions in LUAD.

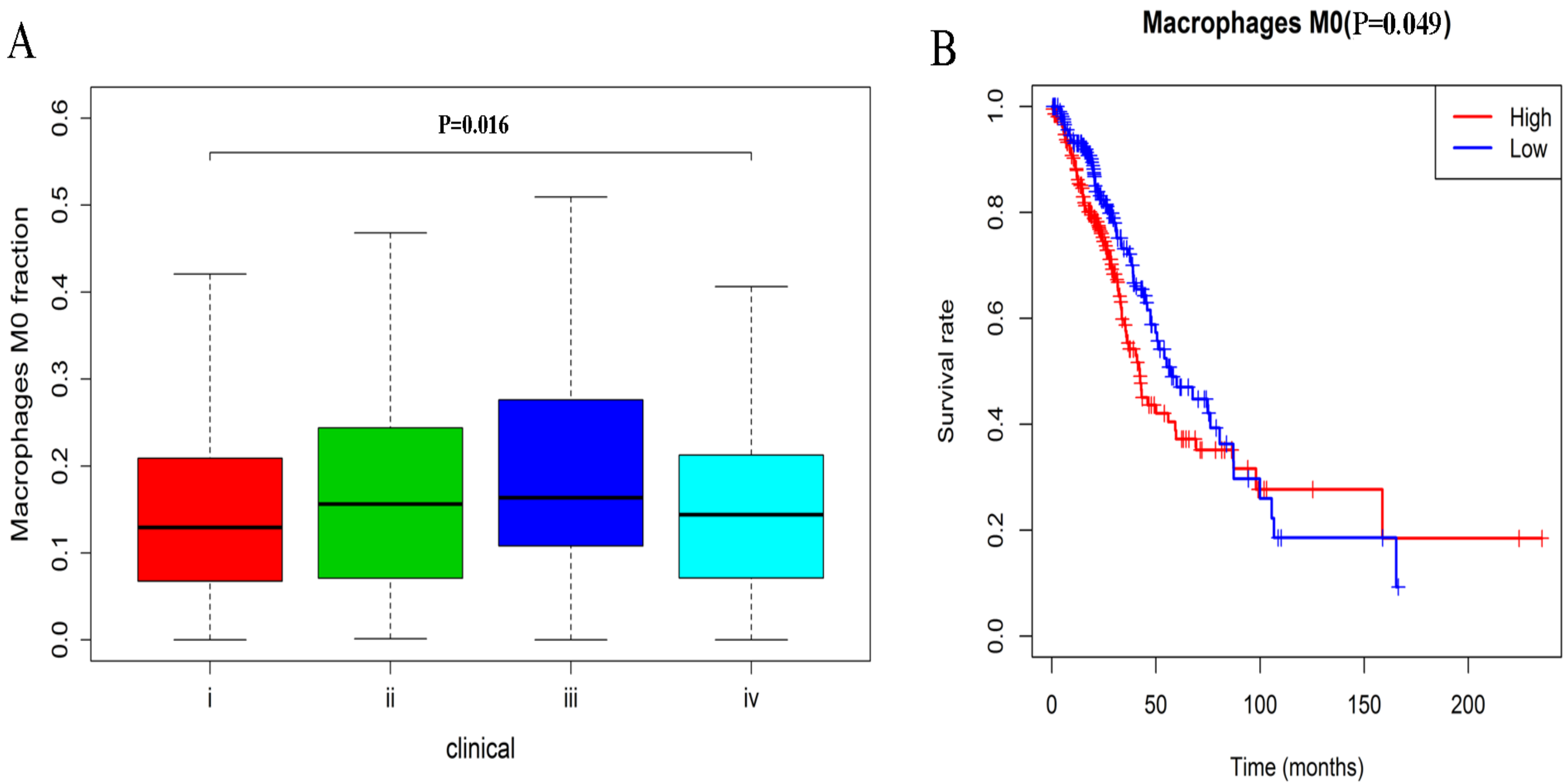

Supplemental Figure2. Associations between tumor stages/survival and the abundance of TIICs in LUAD.

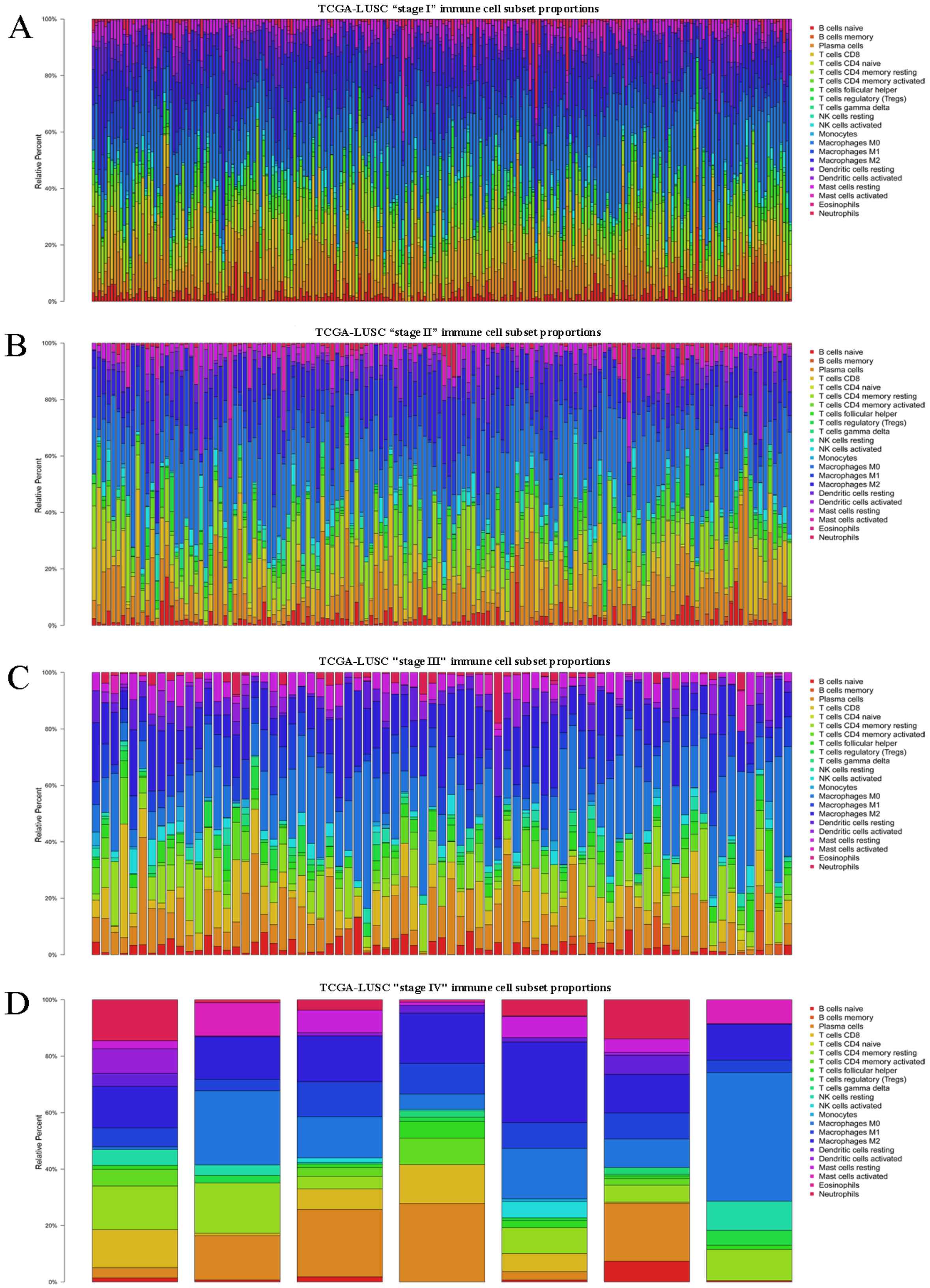

Supplemental Figure 3. The bar charts of immune cell subset proportions in LUSC.

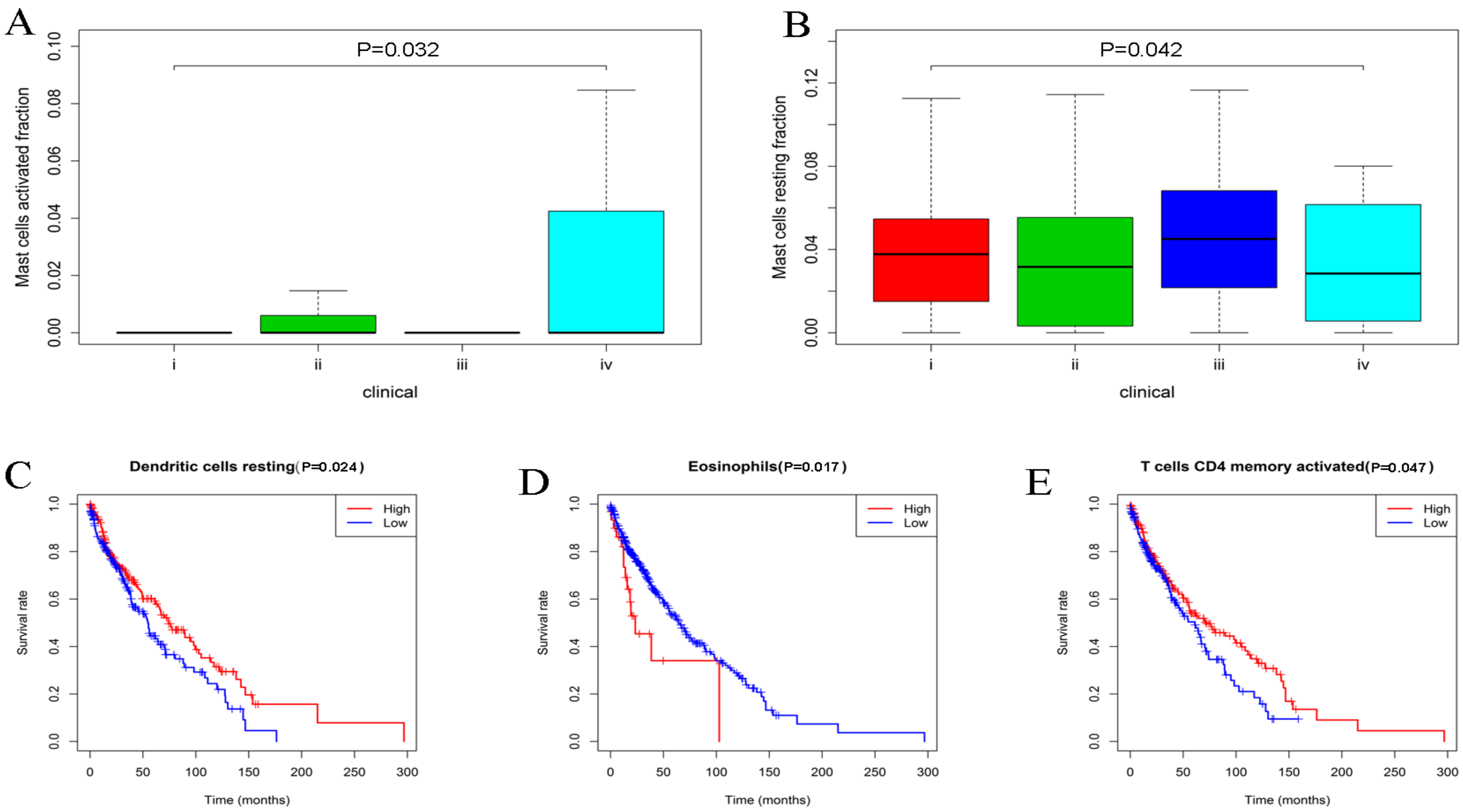

Supplemental Figure 4. Associations between tumor stages/survival and the abundance of TIICs in LUSC.
